# Changes and Challenges in Inpatient Mental Health Care During the First Two High Incidence Phases of the COVID-19 Pandemic in Germany – Results From the COVID Ψ Psychiatry Survey

**DOI:** 10.3389/fpsyt.2022.855040

**Published:** 2022-04-27

**Authors:** Hauke Felix Wiegand, Anna-Lena Bröcker, Mandy Fehr, Niklas Lohmann, Birgit Maicher, Nikolaus Röthke, Mike Rueb, Paula Wessels, Moritz de Greck, Andrea Pfennig, Stefan Unterecker, Oliver Tüscher, Henrik Walter, Peter Falkai, Klaus Lieb, Lars Peer Hölzel, Kristina Adorjan

**Affiliations:** ^1^Department of Psychiatry and Psychotherapy, University Medical Center of the Johannes Gutenberg-University Mainz, Mainz, Germany; ^2^Department of Psychiatry and Psychotherapy, Charité Campus Mitte, Charité Universitätsmedizin Berlin, Corporate Member of Freie Universität Berlin, Humboldt-Universität Zu Berlin, and Berlin Institute of Health, Berlin, Germany; ^3^Department of Psychiatry and Psychotherapy, Carl Gustav Carus University Hospital, Medical Faculty, Technische Universität Dresden, Dresden, Germany; ^4^Department of Psychiatry and Psychotherapy, University Hospital, Ludwig Maximilians University Munich, Munich, Germany; ^5^Department of Psychiatry, Psychosomatics and Psychotherapy, University Hospital of Wurzburg, Wurzburg, Germany; ^6^Department of Psychiatry, Psychosomatics and Psychotherapy, University Hospital of Frankfurt, Frankfurt, Germany; ^7^Oberberg Parkklinik Schlangenbad, Wiesbaden Schlangenbad, Wiesbaden, Germany

**Keywords:** COVID-19, pandemic, mental health care, inpatient care, psychiatry, telemedicine

## Abstract

Psychiatric inpatient treatment, an important pillar of mental health care, is often of longer duration in Germany than in other countries. The COVID-19 pandemic called for infection prevention and control measures and thereby led to shifts in demand and inpatient capacities. The Germany-wide *COVID* Ψ *Psychiatry Survey* surveyed department heads of German psychiatric inpatient institutions. It assessed changes in utilization during the first two high incidence phases of the pandemic (spring 2020 and winter 2020/21) and also consequences for care, telemedicine experiences, hygiene measures, treatment of patients with mental illness and co-occuring SARS-CoV-2, and coercive measures in such patients. A total of *n* = 71 psychiatric departments (of 346 contacted) participated in the survey. The results showed a median decrease of inpatient treatment to 80% of 2019 levels and of day hospital treatment to 50% (first phase) and 70% (second phase). Reductions were mainly due to decreases in elective admissions, and emergency admissions remained unchanged or increased in 87% of departments. Utilization was reduced for affective, anxiety, personality, and addiction disorders but appeared roughly unaffected for psychotic disorders. A lack of integration of patients into their living environment, disease exacerbations, loss of contact, and suicide attempts were reported as problems resulting from reduced capacities and insufficient outpatient treatment alternatives. Almost all departments (96%) treated patients with severe mental illness and co-occurring SARS-CoV-2 infection. The majority established special wards and separate areas for (potentially) infectious patients. Telephone and video consultations were found to provide benefits in affective and anxiety disorders. Involuntary admissions of persons without mental illness because of infection protection law violations were reported by 6% of the hospitals. The survey showed high adaptability of psychiatric departments, which managed large capacity shifts and introduced new services for infectious patients, which include telemedicine services. However, the pandemic exacerbated some of the shortcomings of the German mental health system: Avoidable complications resulted from the lack of cooperation and integrated care sequences between in- and outpatient sectors and limited options for psychiatric hospitals to provide outpatient services. Preventive approaches to handle comparable pandemic situations in the future should focus on addressing these shortcomings.

## Introduction

Psychiatric inpatient treatment, an important pillar of mental health care, often is of longer duration in Germany than in other countries (1–3). The COVID-19 pandemic represented a major challenge for this inpatient care system in several respects: Hygiene and infection control measures had to be implemented to protect hospitalized patients from COVID-19 infections, and capacities had to be provided for the challenging population with co-occurring severe mental illness (SMI) and SARS-CoV-2 infection, often at the expense of regular services. Large pandemic-related shifts in utilization of mental health services have been reported from other countries, such as Italy and the UK (4–6). The reported effects of the pandemic included both limitations in capacities in healthcare systems that had reached their limits by treating SARS-CoV-2 infections and patients who either avoided services because they were worried about infection or used them more often because of the psychosocial burden of the pandemic and accompanying lockdown measures (4–6). In the German mental health system, these shifts occurred within a mental health billing system whose incentive structure rewards full utilization and in which reduced utilization is punished by financial losses and threats of long-term capacity reductions. These ongoing challenges are happening against the background of a higher risk for severe COVID-19 outcomes among people with pre-existing SMI (7) and increase in distress and thereby risk of mental illness among risk groups in the general population (8) and populations with pre-existing mental illness (9).

A first survey of 38 hospitals and departments of psychiatry and psychotherapy in Germany in the first high incidence phase of the pandemic, spring 2020, reported a significant reduction in capacities, such as a reduction in mean occupancy of nearly 40%. A total of 84% of the institutions reported that they had established special wards or spaces for providing care for patients with SMI and comorbid SARS-CoV-2 infection, but that these areas were not used much because the first high incidence phase in Germany was not as severe as expected (10). The studies of routine data from a German psychiatric hospital network reported an overall decrease in emergency hospital admissions during this phase but an increase in the proportion of involuntary and urgent admissions (11, 12). The evidence also hints at significant reductions in utilization in the German outpatient mental healthcare system during the first wave of the pandemic: For example, in March 2020, the number of individual psychotherapy cases decreased by 23%, and in April 2020, the number of psychiatric treatment cases, as measured by case-based lump sum payments, decreased by 30% and the number of group therapy cases decreased by as much as 60% (13).

The above findings raised questions of how continuity of care was maintained for patients affected by the service reductions, especially in the inpatient sector, and how the system reacted in the subsequent high incidence phase of the pandemic in winter 2020/2021, when COVID-19 incidences were much higher than in spring 2020 (14). Therefore, the *COVID* Ψ *Psychiatry Survey* examined and compared changes in capacities and utilization in Germany during the first two high incidence phases of the COVID-19 pandemic in spring 2020 and winter 2020–2021; the consequences of the reduced capacities on care and the financial situation of healthcare providers; experiences with hygiene and protective measures, the treatment of patients with co-occurring SMI and SARS-CoV-2 infection, and telemedicine services in mental health care; coercive measures in patients with SARS-CoV-2-positive; and SARS-CoV-2 infections among mental healthcare employees.

## Methods

Between March and June 2021, we used the information in the 2018 Directory of Hospitals from the Federal Statistical Office to contact 346 hospitals and departments of psychiatry and psychotherapy by email and invite them to participate in the survey. Hospitals and departments from all postal code regions in Germany (1–9) were included. The directors or head physicians of 71 of the 346 (21%) psychiatric-psychotherapeutic hospitals and departments took part in the survey. They were allowed to choose between either filling in an online survey (*n* = 39; 55%) directly or being guided through the questions during an online video call (*n* = 32; 45%). The questionnaire was anonymous and identical in both scenarios. All answers were entered in LymeSurvey^®^ on the data-protected servers of Mainz University Medicine. An English translation of the original German questions is provided in Supplementary Table S1.

The Data Protection Officer at Mainz University Medicine confirmed that no data protection vote was required because no personal data were processed. The ethics commission of the Ludwig Maximilians University Munich had already approved the first study (10), that is, the anonymous survey of hospitals or departments, in which no personal or individual patient data were collected.

The collected data were analyzed on a descriptive level with R 4.0.1 for MAC OS X and Microsoft Excel for Mac 16.16.27. Medians and interquartile ranges (IQR) were calculated because distributions were skewed and to deal with outliers. Percentages refer to the total number of responses to the respective question. Free text responses were examined by qualitative content analysis according to Mayring (15). Answers were grouped according to their content context and categorized independently by two authors (MF and LPH); conflicting categorizations were discussed by three authors (MF, LPH, and HFW), who reached a consensus.

## Results

### Profile of Hospitals and Departments

A total of *n* = 71 hospitals and departments of psychiatry and psychotherapy took part in the survey. Among the respondents, 82% (*n* = 58) offered inpatient treatment; 80% (*n* = 57), day hospital treatment; and 82% (*n* = 58), outpatient treatment.

### Treatment Capacities and Utilization

**Figure 1:**
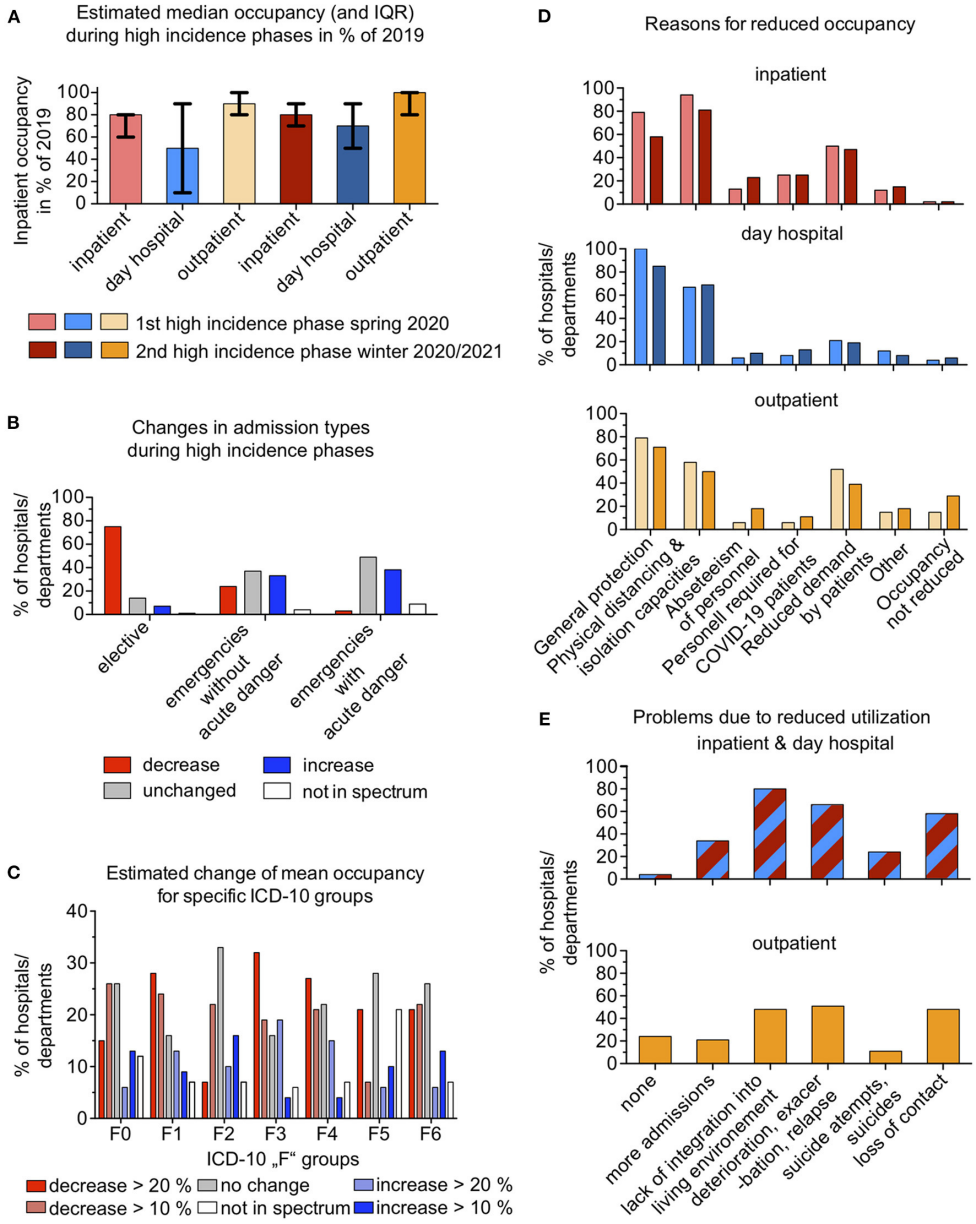
**(A)** shows medians and interquartile ranges of the estimated occupancy during the high incidence phases of the COVID-19 pandemic in Germany in spring 2020 and winter 2020/2021 as a percentage of the occupancy during the equivalent periods in 2019. Red, inpatient occupancy; blue, day hospital occupancy; yellow, occupancy in hospital outpatient clinics. **(B)** shows changes in admission types during both high incidence phases (spring 2020 and winter 2020/2021) compared with the equivalent periods in 2019. Red, percentage of departments that reported a decrease in the mentioned admission type; gray, percentage of departments that reported no change in the mentioned admission type; blue, percentage of departments that reported an increase in the mentioned admission type. **(C)** shows the estimated change of mean occupancy for specific ICD-10 groups in both high incidence phases. F0, organic mental disorders; F1, addiction disorders; F2, psychoses; F3, affective disorders; F4, neurotic, stress-related, and somatoform disorders; F5, eating disorders; F6, personality disorders. **(D)** shows selected reasons for a reduced occupancy during the two high incidence phases of the pandemic. For other reasons, refer to Supplementary Table S2. Red, inpatient care; blue, day hospital care; yellow, hospital-based outpatient care. Lighter colors, first high incidence phase in spring 2020; darker colors, second high incidence phase in winter 2020/2021. **(E)** shows selected problems due to a reduced utilization of services during the two high incidence phases. For other reasons, refer to Supplementary Table S2. Red/blue, inpatient and day hospital care; yellow, hospital-based outpatient care.

In both the first (spring 2020) and second (winter 2020/2021) high incidence phases, the estimated inpatient occupancy was reduced to a median of 80% (IQR 20%) of the pre-pandemic periods in spring 2019 and winter 2019–2020, respectively. Day hospital treatment was reduced to a median of 50% (IQR 75%) of the respective 2019 level in the first high incidence phase and to a median of 70% (IQR 40%) in the second such phase. Outpatient treatment at the hospitals or departments was reduced to a median of 90% (IQR 20%) in the first high incidence phase but was not lower than in 2019 (median, 100%; IQR, 20%) during the second such phase (Figure 1A). When asked about changes in types of admission, 75% (*n* = 52) of the hospitals or departments reported a decrease in elective admissions; 14% (*n* = 10), no change; and 7% (*n* = 5), an increase. A total of 24% (*n* = 17) reported a decrease in emergency admissions without an acute risk; 37% (*n* = 26), no change; and 33% (*n* = 23), an increase. A few (4%, *n* = 3) reported that they did not offer these types of admissions. Among all participating institutions, 3% (*n* = 2) indicated a decrease in emergency admissions with an acute risk; 49% (*n* = 34), no change; and 38% (*n* = 26), an increase. Some institutions (9%, *n* = 6) reported not offering these admissions (Figure 1B).

In terms of diagnosis groups, decreases of more than 20% compared with the same periods in 2019 were reported by 32% (*n* = 22) of the hospitals or departments for affective disorders (ICD-10 F3); by 28% (*n* = 19) for addictive disorders (ICD-10 F1); by 27% (*n* = 18) for neurotic, stress-related, and somatoform disorders (ICD-10 F4); and by 21% (*n*=14) each for personality (ICD-10 F6) and eating disorders (ICD-10 F5). Only 15% (*n* = 10) of the hospitals or departments reported decreases of **>**20% for organic mental disorders (ICD-10 F0), and only 7% (*n* = 5), for schizophrenic psychoses (ICD-10 F2). Increases **>**20% were reported by 16% (*n* = 11) of hospitals or departments for schizophrenic psychoses, by 13% (*n* = 9) for organic mental disorders, by 13% (*n* = 9) for personality disorders, and by 10% (*n* = 14) for eating disorders (refer to Figure 1C for additional results).

Among the hospitals or departments that reported reduced inpatient treatment occupancy, the associated reasons were given as follows: to allow for isolation capacities and to maintain physical distancing, 94% (*n* = 49) in the first high incidence phase and 81% (*n* = 43) in the second; as a general protective measure, 79 (*n* = 41) and 58% (*n* = 31); because of reduced patient demand, 50 (*n* = 26) and 47% (*n* = 25); because staff had to be assigned to caring for patients with COVID-19, 25 (*n* = 13) and 25% (*n* = 13); because of COVID-19 related staff shortages, 13% (*n* = 7) and 23% (*n* = 12); and for other reasons, 12 (*n* = 6) and 15% (*n* = 8). Regarding reductions in day hospital treatment, 100% *(n* = 52) in the first high incidence phase and 85% (*n* = 41) in the second stated that the reductions were a general protective measure; 67 (*n* = 35) and 69% (*n* = 33), to maintain physical distancing; 21 (*n* = 11) and 19% (*n* = 9), due to reduced patient demand; 8 (*n* = 4) and 13% (*n* = 6), because staff had to be assigned to caring for patients with COVID-19; 6 (*n* = 3) and 10% (*n* = 5), due to COVID-19–related staff shortages; and 12 (*n* = 6) and 8% (*n* = 4), for other reasons. Regarding outpatient treatment reductions, 79% (*n* = 26) in the first high incidence phase and 71% (*n* = 20) in the second reported that it was a general protective measure; 58 (*n* = 19) and 50% (*n* = 14), to maintain social distancing; 52 (*n* = 17) and 39% (*n* = 11), due to reduced patient demand; 6 (*n* = 2) and 11% (*n* = 3), because staff had to be assigned to caring for patients with COVID-19; 6 (*n* = 2) and 18% (*n* = 5), due to COVID-19-related staff absences; and 15 (n = 5) and 18% (*n* = 5), for other reasons (Figure 1D).

When asked about the difficulties due to reduced inpatient and day hospital capacity and occupancy, 80% (*n* = 57) reported a lack of integration of patients into their living environment at the end of treatment; 66% (*n* = 47), deteriorations, exacerbations, and relapses; 58% (*n* = 41), loss of contact with patients; 34% (*n* = 24), increased admissions after the pandemic waves; and 24% (*n* = 17), suicide attempts and suicides. A total of 4% (*n* = 3) reported no difficulties. As a consequence of limited outpatient care, 51% (*n* = 36) perceived deteriorations, exacerbations, and relapses; 48% (*n* = 34) each, loss of contact with patients and a lack of integration of patients into their living environment at the end of treatment; 24% (*n* = 17), no difficulties; 21% (*n* = 15), increased admissions after the high incidence phases; and 11% (*n* = 8), suicide attempts and suicides (Figure 1E). As regards patients who were not admitted or were discharged prematurely because of the capacity reductions, 84% (*n* = 53) of the hospitals or departments reported that the patients were treated in their own outpatient clinic instead; 62% (*n* = 39), *via* telemedicine services; 40% (*n* = 25), by outpatient psychiatrists and neurologists in private practice; and 30% (*n* = 19), by psychotherapists in private practice. In the free text responses, participants mentioned a lack of compensatory outpatient sector options; a lack of coordination between in- and outpatient sectors; and problems due to restricted outpatient psychosocial services in the pandemic, which caused a higher burden for patients with SMI and, as a consequence, more or repetitive acute inpatient admissions (refer to Supplementary Table S1, questions 5b, 7, 8, and 34).

Among the respondents, 36% (*n* = 25) reported offering home treatment (“stationsäquivalente Behandlung”). Of these, 72% (*n* = 18) reported continuing to offer it; 9% (*n* = 6), having paused it as a protective measure for patients and staff; and 1% (*n* = 1), stopping it at the request of patients. In the free text responses, participants found home treatment to be a good alternative to relieve the restricted inpatient services (refer to Supplementary Table S1, question 34).

Concerns about financial losses because of the pandemic-related capacity reductions were reported by 72% (*n* = 51) of the departments; in 14% (*n* = 7) of these departments, the concerns were due to the lack of compensation payments (i.e., of payments for beds that were unoccupied due to infection protection measures) in the first high incidence phase and in 96% (*n* = 49), due to the lack of such payments in the second high incidence phase. In the free text responses, several participants complained about a lack of or uncertainty about compensation payments for unoccupied beds. However, some participants considered the initial compensation payments in the first high incidence phase as a disincentive that resulted in larger capacity reductions than necessary (refer to Supplementary Table S1, question 34).

### Telemedicine

**Figure 2:**
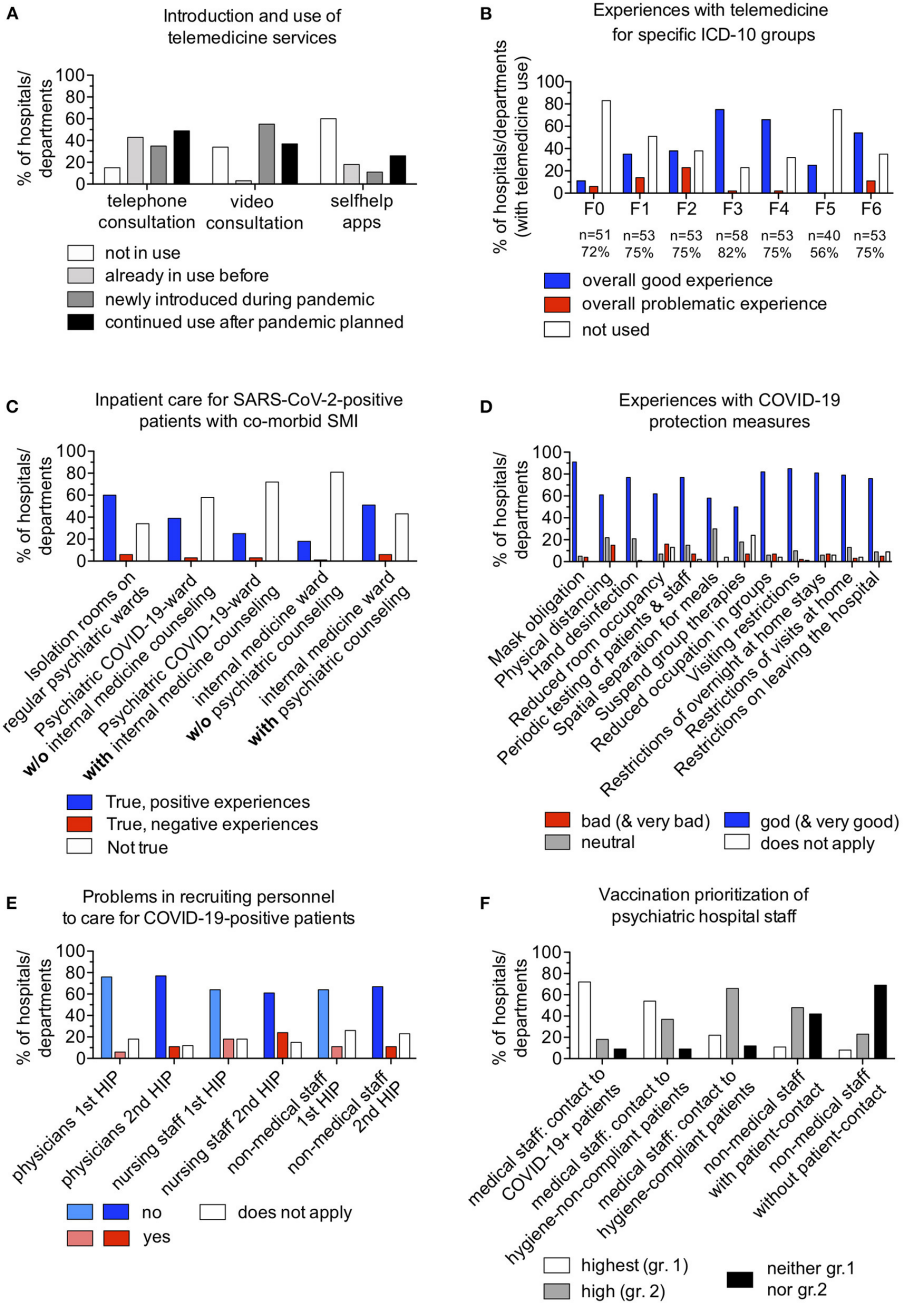
**(A)** shows findings about the introduction and use of telemedicine services during the pandemic. **(B)** shows experiences with telemedicine services for specific ICD-10 groups. F0, organic mental disorders; F1, addiction disorders; F2, psychoses; F3, affective disorders; F4, neurotic, stress-related and somatoform disorders; F5, eating disorders; F6, personality disorders. The n-numbers show the number of respondents and the percentages of the total number of participants in the individual groups. Blue, overall good experience within the diagnostic group; red, overall problematic experience within the diagnostic group; white, services not used for this group. **(C)** shows the spatial and internal medicine co-treatment arrangements for patients with severe mental illness and co-occurring SARS-CoV-2 infection. w/o, without. **(D)** shows experiences with specific COVID-19 protection measures. **(E)** shows problems in recruiting personnel for the care of patients with COVID-19-positive. HIP, high incidence phase. Blue, no problems; red, problems in recruiting personnel. Lighter colors, first high incidence phase in spring 2020; darker colors, second high incidence phase in winter 2020/2021. **(F)** shows COVID-19 vaccination prioritization of mental health hospital staff during winter and spring 2021. The categorization is related to the six vaccination priority groups that were defined by federal authorities and determined the temporal order of vaccine distribution.

Telephone consultation services were used by 43% (*n* = 28) of the departments before the pandemic, 25% (*n* = 23) newly introduced them during the pandemic, and 49% (*n* = 32) planned to continue using them after the pandemic. Video consultation services were used by 3% (*n* = 2) before the pandemic, 55% (*n* = 36) newly introduced them in the pandemic, and 37% (*n* = 24) planned to continue using them after the pandemic. Self-help applications were used by 28% (*n* = 12) of hospitals and departments before the pandemic, 11% (*n* = 7) newly introduced them in the pandemic, and 26% (*n* = 17) planned to continue using them after the pandemic (Figure 2A). Next, we evaluated the use and experience with telemedicine services in specific diagnostic groups: For addictive disorders (ICD-10 F1), the question was answered by 75% (*n* = 53) of the participants, 49% of which used telemedicine services for patients with this disorder; 35% (*n* = 23) reported overall good experiences, and 14% (*n* = 9), problematic experiences. For psychoses (ICD-10 F2), the question was answered also by 75% (*n* = 53), 62% of which used telemedicine services for patients with this disorder; 39% (*n* = 25) reported overall good experiences, and 23% (*n* = 15), problematic experiences. For affective disorders (ICD-10 F3), the question was answered by 82% (*n* = 58) of the participants, 77% of which used telemedicine services for patients with this disorder; 75% (*n* = 49) reported overall good experiences, and 2% (*n* = 1), problematic experiences. For neurotic, stress-related, and somatoform disorders (ICD-10 F4), the question was answered by 75% (n = 53) of the participants, 68% of which used telemedicine services for patients with this disorder; 66% (*n* = 43) reported overall good experiences, and 2% (*n* = 1), problematic experiences. For personality disorders (ICD-10 F6), the question was answered by 65% (*n* = 53) of the participants, 65% of which used telemedicine services for patients with these disorders; 54% (*n* = 35) reported overall good experiences, and 11% (*n* = 7), problematic experiences. Only *n* = 11 participants reported using telemedicine services for organic mental disorders (ICD-10 F0) and only *n* = 16 for eating disorders (ICD-10 F5), so interpretations of the experiences within these disorder groups are limited (Figure 2B). In the free text responses, several participants highlighted that the pandemic represented a good opportunity to flexibly and unbureaucratically install a telemedicine infrastructure. Furthermore, they remarked that this infrastructure could be used to simplify internal conferences, education, and training. However, others mentioned problems with providing telemedicine services to patients with SMI and structural deficits because these patients often do not have access to the required technical prerequisites (refer to Supplementary Table S1, questions 8 and 34).

### Capacities for Patients With SARS-CoV-2-Positive With SMI

Next, we asked about settings, conditions, and care experiences with patients with SMI and comorbid SARS-CoV-2 infection. Such patients were treated by 96% (*n* = 64) of the departments: 49% (*n* = 33) treated 1 to 10 patients; 19% (*n* = 13), 11 to 20; and 27% (*n* = 18), more than 20. The outbreaks of SARS-CoV-2 infection on wards were reported by 57% (*n* = 38). Regarding infrastructure for treatment of patients with SARS-CoV-2–positive, 65% (*n* = 36) of the departments reported having an oxygen supply; 58% (*n* = 32), rooms that could be used as an “airlock”; 35% (*n* = 19), a monitor; and 33% (*n* = 18), none of the aforementioned infrastructure. The question was not answered by 5% (*n* = 3). Standardized operating procedures (SOPs) for the treatment of psychiatric patients with COVID-19 were available in 43% (*n* = 29) of the departments, and 54% (*n* = 36) reported that there was a specialized COVID-19 ward somewhere in the hospital. Care for patients with SARS-CoV-2-positive with SMI was organized in isolation rooms on regular psychiatric wards in 66% (*n* = 44) of the departments, 9% (*n* = 4) of which reported negative experiences with this arrangement. A dedicated COVID-19 ward in the psychiatric unit without internal medicine co-treatment was present in 42% (*n* = 28) of the departments, 7% (*n* = 2) of whom reported negative experiences; a dedicated COVID-19 ward in the psychiatric department with internal medicine co-treatment was reported by 28% (*n* = 19) of the departments, 11% (*n* = 2) of which reported negative experiences; an internal medicine ward with consultative psychiatric co-treatment was present in 57% (*n* = 38) of the hospitals or departments, 11% (*n* = 4) of which reported negative experiences; and an internal medicine ward without psychiatric consultation for this group of patients existed in 19% (*n* = 13) of the facilities, 8% (*n* = 1) of which reported negative experiences (Figure 2C). Internal medicine consultation treatment for patients with SMI and comorbid COVID-19 was available on site in 52% (*n* = 35) of the hospitals or departments, and internal medicine telephone or video consultation was available in 43% (*n* = 29). Transfers from psychiatric wards to internal medicine wards occurred at 82% (*n* = 55) of the departments, with 40% (*n* = 22) reporting difficulties. Among those reporting difficulties, 82% (*n* = 18) stated that the internal medicine wards had reservations and fears about mentally ill patients; 64% (*n* = 14), that the internal medicine staff had questioned the assessment of patients as requiring critical medical care; 45% (*n* = 10), that the internal medicine ward or hospital had no free capacity; and 41% (*n* = 9), that the ability to provide medical care in psychiatry had been overestimated by the internal medicine departments. In the free text responses, several participants highlighted that cooperation and interdisciplinarity with internal medicine had improved as a consequence of the pandemic. However, multiple participants mentioned constructional limitations of many departments' buildings, which posed a great challenge to creating capacities for highly infectious patients (refer to Supplementary Table S1, question 34).

### SARS-CoV-2-Positive Patients and Coercion Measures

Among the hospitals or departments, 58% (*n* = 39) reported that patients with SARS-CoV-2-positive with SMI had been admitted involuntarily under the standard mental health acts for endangerment of self or others (“Landesgesetze über Hilfen bei psychisch Erkrankungen PsychKHG/PsychKG” or “Bundesgesetzbuch BGB”). Furthermore, in 25% (*n* = 17) of the hospitals or departments, patients with SARS-CoV-2 infection had been admitted involuntarily under infection protection laws (Infektionsschutzgesetz IfSchG). In 19% (*n* = 13) of the hospitals or departments, besides SARS-CoV-2 infection, the patients had mental illness. However, in 6% (*n* = 4) of the hospitals or departments, patients with SARS-CoV-2 infection without prominent symptoms of mental illness were admitted involuntarily to psychiatric hospitals or departments. Additionally, 13% (*n* = 9) of the hospitals or departments reported that public authorities had presented people without SMI because they had disregarded hygiene rules but that the people had not been admitted.

### Hygiene, Infection Protection, and Containment Measures

The next set of questions was about the experiences with hygiene, infection protection, and containment measures. A “pandemic plan” with organizational measures for the case of a pandemic was in place before the start of the pandemic in 45% (*n* = 30) of the hospitals or departments, 46% (*n* = 31) implemented the plan during the pandemic, and 9% (*n* = 6) had not implemented it at the time of the survey. We further asked how hygiene and protection measures could be implemented in the context of a mental health hospitals or departments, that is, we asked about mandatory masks (that cover the nose and mouth), social distancing, reduced room occupancy, periodic testing of staff and patients, spatial separation during meals, reduced size of therapy groups, visiting restrictions, and restrictions of overnight stays at homes and home visits for hospitalized patients. Overall, most respondents rated the implementation of these hygiene and protection measures as “good or very good.” However, fewer hospitals or departments rated the implementation of the following items as such: social distancing (63%, *n* = 42), reduced room occupancy (63%, *n* = 42), spatial separation during meals (58%, *n* = 39), and suspension of group therapies (51%, *n* = 34). For additional results, refer to Figure 2D. In the free text responses, several participants noted some aspects as being problematic: hygiene regulatory requirements being rapidly changed by regional authorities, a lack of protection gear at the beginning of the pandemic, and the large increases in the time and personnel resources required for organizing testing and hygiene measures. Respondents highlighted the importance of immediately testing newly admitted patients, having a crisis management group that met frequently, making one person responsible and available for questions about hygiene and protection measures, and frequently communicating measures and changes to personnel and patients (refer to Supplementary Table S1, question 34).

### Personnel, Absenteeism, and Vaccination Prioritization

SARS-CoV-2 infections among staff occurred at 89% *(n* = 59) of the hospitals or departments, with median maximum staff absences of 10% (range, 1 to 50%). The hospitals or departments encountered problems recruiting various types of personnel to care for patients with SARS-CoV-2-positive in the first and second high incidence phases, as follows: difficulties recruiting physicians, 6% (*n* = 4) and 11% (*n* = 7), respectively; difficulties recruiting nursing staff, 18% (*n* = 12) and 24% (*n* = 16), respectively; and difficulties recruiting non-medical staff, 11% (*n* = 7) in both phases, (Figure 2E). Increased absenteeism among staff caring for patients with SARS-CoV-2–positive at the hospitals or departments occurred in the first and second high incidence phases, as follows: among physicians, 11% (*n* = 7) and 12% (*n* = 8), respectively; among nurses, 29% (*n* = 19) and 27% (*n* = 18), respectively; and among non-medical staff, 14% (*n* = 9) in both phases. In the free text responses, several participants reported absenteeism among nursing and non-medical staff due to anxieties about contagion risk, and they highlighted the real, significantly elevated risk for medical staff on acute psychiatry wards, where disorganized, potentially patients with COVID-19-positive were not able to comply with hygiene and protection measures. As a good practice, several hospitals or departments introduced additional supervision and support offerings for medical personnel. Several participants described the overall commitment of the personnel as very good. However, some reported that the personnel in day hospitals or elective psychotherapy units interpreted the closure of the units as a devaluation of their work (refer to Supplementary Table S1, question 34).

In Germany, federal authorities defined six vaccination priority groups (16) and vaccines were distributed accordingly in the order of priority. However, the assignment of mental healthcare workers to these groups was handled quite differently depending on the region and employer, as highlighted by the survey: Healthcare personnel (nurses, physicians, therapists) who had contact with patients with COVID-19-positive were in the highest priority group in 72% (*n* = 47) of the hospitals or departments, in the high priority group in 18% (*n* = 12), and in neither of those in 9%. Healthcare workers who had contact with patients who failed to comply with protective measures were in the highest priority group in 54% (*n* = 35) of hospitals or departments, in the high priority group in 37% (*n* = 24), and in lower categories in 9% (*n* = 6). Healthcare workers who had contact with patients who were able to comply with the protective measures were in the highest priority group in 22% (*n* = 14), in the high priority group in 66% (*n* = 43), and in the residual categories in 12% (*n* = 8). Non-medical staff who had patient contact were in the highest priority group in 11% (*n* = 7) of hospitals or facilities, in the high priority group in 48% (*n* = 31), and in a lower category in 42% (*n* = 27; Figure 2F).

## Discussion

### Changes in Utilization

The *COVID* Ψ *Psychiatry Survey* showed that, compared with the 2019 levels in the same periods, during the COVID-19 pandemic's high incidence phases in spring 2020 and winter 2020–2021, inpatient occupancy at psychiatric hospitals and departments in Germany was reduced by approximately a quarter, and day hospital occupancy, by half. Although this applied to a wide range of diagnoses (e.g., affective, addictive, anxiety/OCD/stress-related/somatoform, personality, and eating disorders), results for individuals with psychosis or organic mental disorders were mixed, with some departments reporting increases and some decreases in occupancy. Even though elective admissions were reduced, many departments reported increases in emergency admissions because of endangerment to self or others. The most common reasons for an overall reduced occupancy were to provide capacities for infective patients and to allow for physical distancing and general protection measures. Only half of the hospitals and departments reported reduced demand as a reason, and only a quarter, the assignment of staff to COVID-19 wards. To compensate for capacity reductions, the hospitals or departments reported attempting to treat patients in their own outpatient clinics and by telemedicine services. Approximately one-third of the departments reported that patients who were not admitted received treatment in the outpatient system instead.

These reported decreases in inpatient care during the pandemic's high incidence phases in spring 2020 and winter 2020–2021 represent substantial disruptions of normal services and seem to have had at least partially problematic consequences. The service reductions seemed to be comparable to those in outpatient and emergency services in other countries, as suggested by reports from the UK, Spain, and the US (4–6, 17, 18). However, this heterogenous patchwork of reports shows that more research is urgently needed on the disruptive effects of the pandemic on mental health services.

### Patients With SMI and SARS-CoV-2 Infection

Nearly all departments treated patients with SMI and co-occurring SARS-CoV-2 infection. For this population, they used different settings: Approximately two-thirds used isolation rooms on regular psychiatric wards, and two-thirds used dedicated COVID-19 wards. In addition, many patients were treated on internal medicine wards that had psychiatric consultation services. The cooperation with internal medicine was rated as mostly good, but transfers were sometimes difficult, especially because of a fear of patients with mental illness, a different assessment of patients as requiring critical medical care and of the ability to provide psychiatric care, and a lack of capacities in internal medicine. To avoid such issues in the event of future crisis situations, it might be helpful to establish regular meetings between staff in internal medicine and psychiatry to create a trusting and reliable basis for crises, and some hospitals are already taking such an approach. In addition, it appears to be essential to continually destigmatize and educate about mental illnesses in neighboring disciplines.

### A Flexible and Adaptive Good-Practice Response

On the one hand, the results show how, overall, the psychiatric hospitals or departments and their staff adapted flexibly to the pandemic, with nearly all of the facilities providing opportunities for treating highly infectious patients with SARS-CoV-2-positive, who were often unable to follow hygiene rules because of their mental illness. This flexibility is especially remarkable in light of the limited spatial and technical resources for the treatment of highly infectious patients and of an incentive structure that financially “punishes” facilities for having unoccupied beds. The estimated financial losses may indicate that most departments prioritized patient safety over economic interests—an encouraging sign from the perspective of medical ethics. The hygiene and protective measures queried in our survey were mainly evaluated positively and appear to have proven effective. Furthermore, the free text responses revealed many creative ideas that were used for organizing mental health care safely during the pandemic.

### Lack of Cooperation and Coordination Between in- and Outpatient Sectors

On the other hand, the survey revealed several problematic areas: Participants saw connections between capacity reductions, especially of day hospital and elective inpatient services, and reports about deteriorations, exacerbations, difficulties in organizing the integration of patients into their living environment after discharge, increases in acute admissions and sometimes in suicide attempts. Even if the given results are descriptive and do not allow for causal interpretations, it can be assumed that the pandemic exacerbated pre-existing deficits of the German mental healthcare system, namely, the lack of integration, coordination, and governance between the in- and outpatient sectors. Reductions in inpatient capacity could not be fully covered by outpatient services, and on a regional level, awareness and oversight of these needs probably did not even exist.

For affective disorders, addiction, anxiety, and personality disorders, a short-term partial replacement of inpatient treatment seemed to be possible through outpatient and telemedical services. However, in most federal states in Germany, hospital-based outpatient services (“Psychiatrische Institutsambulanzen” [PIA]) usually receive only quarterly flat-rate payments that do not allow for treatment for SMI at a frequency that complies with the guideline recommendations [e.g., (19, 20)]. As a consequence, in these disorder groups, the departments' outpatient and telemedicine services must be viewed only as a short-term emergency solution and not as a fully-fledged alternative. In the case of psychoses and organic mental disorders, the changes during the pandemic were more heterogeneous and reductions less pronounced. At the same time, the survey showed that telemedicine solutions were often not considered to be an alternative for these conditions [for a similar conclusion, see (18)]. To provide adequate care, particularly for these groups, and to minimize inpatient stays, which carry a higher risk of infection, the billing system should consider alternative care models, such as outreach treatments by hospital staff in a non-bureaucratic and more flexible manner. Because there is insufficient evidence to support the use of self-help apps for patients with SMI (21), it was appropriate that they played only a minor role in supplementing but not replacing services.

### A Lack of Real-Time Data Access for Monitoring Utilization and Sequences of Care

As an important pillar in the integrated framework for maintaining services during the COVID-19 pandemic, the World Health Organization recommended systematic monitoring of essential health services through regular tracking, analysis, and reporting on healthcare utilization and delivery (22). Routine data on utilization and sequences of care in in- and outpatient sectors exist also in Germany, but unfortunately, they are not accessible in a timely manner for either policy advice or for research purposes (23). This lack of data severely limits the possibility to base healthcare policy measures on data and evidence, even more so during a pandemic, where rapid action is necessary. The *COVID* Ψ *Psychiatry Survey* can paint only a very coarse picture of the mental healthcare situation during the high incidence phases of the COVID-19 pandemic in Germany, unlike reports from the UK or Israel, where research was able to evaluate the effects of healthcare politics decisions in large and representative longitudinal cohorts (5, 17, 24).

### The German Inpatient Mental Health Incentive System in the Pandemic

The reduced occupancy during the second high incidence phase in winter 2020–2021 led to a lack of financial compensation, and most departments estimated that they would incur financial losses in 2020 and 2021. In the German inpatient mental health remuneration—and thereby incentive—system, finding a balance between rewarding maximum utilization and performing evidence-based treatment is a challenge even in non-pandemic times, and low utilization can be penalized in subsequent years. Therefore, we would like to emphasize that reductions in occupancy during the pandemic were related to COVID-19-related protection and containment measures and thereby patient safety. The reported negative consequences of reduced services demonstrate that reductions in inpatient capacities can succeed only if regionally adequate and accessible outpatient alternatives exist. Otherwise, financial punishments for reduced occupancy are problematic for patients, whose quality of care might be endangered, and employees, who already shouldered the load of the pandemic-related burden and dangers.

### Vaccination Prioritization for Mental Healthcare Workers

Nearly all departments treated the patients with SARS-CoV-2-positive and experienced SARS-CoV-2 infections among medical personnel. Staff absenteeism and difficulties in recruiting staff to care for patients with COVID-19 were especially prevalent among nursing staff during the high incidence phase in winter 2020–2021. In this respect, the survey's finding is concerning: In nearly half of the departments, healthcare workers who had contact who had contact with potentially COVID-19-positive patients and with patients who were unable to comply with hygiene measures because of their illness were not in the highest priority vaccination group. The associated reasons are unknown, but stigma and a lack of knowledge about the difficulties and conditions of treating disorganized mentally ill patients with comorbid SARS-CoV-2 infection in mental health institutions might play a role. The hospitals or departments and professional societies should discuss this finding with those responsible in politics and administrations.

### Involuntary Admissions to Mental Healthcare Institutions of People With and Without SMI and the Risk of Stigma

This study results about involuntary admissions of persons with SARS-CoV-2 infections with and without SMI highlight how inpatient psychiatry is caught between the conflicting demands of individual treatment and healing, for which a trusting relationship that respects fundamental and human rights is essential (25), and its societal protection mandate, for example, in case of threats to others in the context of SMI. Too strong a focus on the second aspect can jeopardize the first. In this respect, the authors consider it problematic that this study identified several attempts, fortunately only a few of which were successful, to place people *without* SMI in mental healthcare facilities because they had violated infection protection acts. Such measures carry the high risk of increasing stigmatization of the institution of psychiatry and its users and of losing the fragile good of trust among society and the mentally ill, for whose successful treatment we depend precisely on this.

### International Comparison

Similar to this study, studies in several other regions, including the UK, Italy, Spain, Denmark, Switzerland, Australia, Canada, and the US, observed an overall decrease of inpatient admissions but an increase in involuntary admissions and more acute cases (4, 26–35). Most regions reported reduced utilization for affective and anxiety disorders, but often no change for psychotic and sometimes addiction disorders (36, 37). Similarly, despite often much higher COVID-19 incidences, service restrictions were much less pronounced in the second high incidence phase in winter 2020–2021 than in the first such phase in spring 2020. This pattern may reflect a habituation of the system and its users to the pandemic situation. As in this study, other health systems experienced worse outcomes as a consequence of the reduced mental health services (18, 30, 36, 38). Alarmingly, many particularly vulnerable and precarious groups were especially affected (18, 39). Reports from Italy and France describe similar challenges due to a restructuring of services for patients with comorbid SMI and COVID-19 (40, 41). In many regions, telemedicine was used as a replacement, and the respective experiences were either positive, especially in affective and anxiety disorders, or not so positive, for example, in schizophrenia (18, 40, 42). Another interesting aspect is that absenteeism and distress levels were comparably high among mental healthcare professionals and somatic health professionals (43) but were not so present in the public debate.

### Limitations

A major limitation of this study is the survey format. First, the survey targeted only directors or head physicians of the psychiatric hospitals and departments, so it was biased toward their views and assessment of the situation, which might differ from the views and assessment of other mental healthcare staff or the views of patients. Second, the occupancy numbers collected as a part of the survey were qualitative, and the authors did not have access to the individual hospital's detailed raw data; therefore, the numbers can be seen only as broad estimates. Furthermore, statements about sequences of care, for example, outpatient follow-up treatment, also are not based on actual data because individual hospitals or departments do not have access to reliable outpatient sector data and only to subjective narratives of rehospitalized patients. In general, survey data on sequences of care are much less precise than, for example, routine data studies and data from pre-specified longitudinal cohorts. Therefore, it is important to bear in mind that the results are purely descriptive. For future studies, it would be interesting to conduct inferential statistical analyses of routine data or defined cohorts, also retrospectively, to explore significant associations with positive and negative impacts on treatment outcome during a pandemic.

Another limitation is the sample of participating hospitals or departments. Approximately 20% of the hospitals and departments of psychiatry and psychotherapy in Germany took part in the survey. Factors contributing to non-participation could have been the relatively large effort required for participation (30–40-min duration of the survey, provision of occupancy figures before the survey, etc.). Because of the very heterogenous characteristics of the departments and hospitals, it was difficult to estimate the representativity of the sample in general. In particular, we cannot rule out either that those hospitals and departments that experienced more severe consequences of the pandemic might not have had the capacities to participate in the survey or that those hospitals and departments where the pandemic did not lead to profound changes were not motivated to participate in a survey on the challenges of the pandemic.

The results of the quantitative analyses are limited by the fact that the survey's main focus was not on the questions that required free text responses. Therefore, not all participants responded to them. Furthermore, only question 34 (refer to questionnaire in Supplementary Table S1) was answered by a sufficient number of participants (*n* = 51; 72%), and only the answers to this question showed a certain degree of theoretical saturation, which is a prerequisite for generalizing results of quantitative studies.

## Conclusions

The *COVID* Ψ *Psychiatry Survey* documented the enormous flexibility and adaptability of mental health inpatient institutions and their employees. The way they managed the challenges of treating highly infectious mentally ill patients, who often have difficulties in adapting to stricter hygienic rules because of their illness, deserves great respect. In particular with regard to the limited resources, the need to maintain routine care and successfully implement newly introduced telemedicine services represented a great challenge. However, the survey also showed the disruptive impact of the pandemic on regular mental health services, where the need for implementing social protection measures and creating specialized services for those with co-occurring SMI and SARS-CoV-2 or unclear infection status often came at the price of disrupting regular treatment sequences. It revealed a need for better coordination and governance and more needs-adapted options for bypassing sector (and thus reimbursement) barriers between in- and outpatient services. Longitudinal, representative, and current data on utilization of service sequences should be made more easily accessible for research and regional governance to enable service disruptions to be detected with more precision and in a timely manner. Last but not least, further international research is needed to reliably document and compare the consequences of the COVID-19 pandemic on mental health systems worldwide.

## Data Availability Statement

The raw data supporting the conclusions of this article will be made available by the authors, without undue reservation. The data can be found here: https://github.com/haukefelixwiegand/COVID-Psy-Psychiatry-Survey.git.

## Author Contributions

HW, OT, PF, KL, LH, and KA designed the study. A-LB, MF, NL, BM, MR, NR, PW, MG, AP, SU, OT, HW, PF, and KL contributed and corrected the manuscript. HW, LH, and KA wrote the manuscript. HW, MF, LH, and KA analyzed the data. A-LB, MF, NL, BM, MR, NR, PW, MG, AP, SU, OT, and HW designed the LymeSurvey and conducted the interviews. All authors contributed to the article and approved the submitted version.

## Funding

This project was realized within the German National Network University Medicines [Netzwerk Universitätsmedizin (NUM)] collaborative project egePan Unimed (Development, Testing and Implementation of regionally adaptive health care structures and processes for pandemic management guided by evidence and led by university medicine). egePan Unimed is funded by the German Federal Ministry of Education and Research (BMBF) as part of the Netzwerk Universitätsmedizin (NUM) initiative (Grant-No.: 01KX2021).

## Conflict of Interest

PF received research support/honoraria for lectures or advisory activities from: Abbott, Boehringer-Ingelheim, Janssen, Essex, Lundbeck, Otsuka, Recordati, Richter, Servier, and Takeda. The remaining authors declare that the research was conducted in the absence of any commercial or financial relationships that could be construed as a potential conflict of interest.

## Publisher's Note

All claims expressed in this article are solely those of the authors and do not necessarily represent those of their affiliated organizations, or those of the publisher, the editors and the reviewers. Any product that may be evaluated in this article, or claim that may be made by its manufacturer, is not guaranteed or endorsed by the publisher.
