# Supplementary material for: Changes and Challenges in Inpatient Mental Health Care During the First Two High Incidence Phases of the COVID-19 Pandemic in Germany – Results From the COVID Ψ Psychiatry Survey
Source: Front Psychiatry. 2022 Apr 27;13:855040. doi: 10.3389/fpsyt.2022.855040 (PMC9091906; doi:10.3389/fpsyt.2022.855040)
Supplement: Supplementary file 1 [file Table_1.DOCX]

| **Supplementary Table 1 – Categories and examples from the qualitative content analysis of the free text answers** | |
| --- | --- |
| **Categories** | **Examples** |
| **Question (5b)** | **What other reasons were there for limiting occupancy?** |
| Closure of day clinic | “First wave: complete closure of day clinic services for approximately 1 month for preventive reasons." |
| Reallocation of capacities to other disciplines | "First wave: closing of an entire psychiatric ward to keep it as reserve capacity for COVID-19 patients of the associated somatic medicine services” |
| Quarantine and hygienic isolation capacities | “One ward was cleared for COVID-19 screening and COVID-19 positive patients (loss: 15 beds)" |
| Reduction of beds per room | “Three- and four-bed rooms had to be converted (two-bed rooms max.)” |
| Political interventions: Change in incentive structures | “Legal requirement by regional ministry of the interior that all elective treatments must be terminated in 04/20” |
| Reductions in clinic-based outpatient treatment | “change to telephone contacts” |
| Social distancing and contact reduction | “Interface problems: closure of addiction outpatient services that only worked online and therefore referred fewer patients for inpatient treatments” |
| **Question (7) - Other** | **What other difficulties were encountered due to reduced inpatient and day-clinic care in the two waves of the pandemic?** |
| Restrictions of treatment continuity between inpatient and outpatient sector | “Follow-up treatment difficult; continuous care in outpatient setting difficult” |
| Deteriorations/exacerbations | “Severity of illnesses (especially psychosis & addiction) increased”  “Increased exacerbation of anxiety disorders” |
| Restrictions due to hygiene measures | “Technical and time efforts for coordination, hygiene concepts, staff training” |
| **Question (8) - Other** | **What other difficulties were encountered due to reduced inpatient and day-clinic care in the two waves of the pandemic?** |
| (Difficulties with) telemedicine | “Availability by telephone was not always given” |
| Lack of outpatient care | “Social isolation due to the discontinuation of outpatient groups” |
| Deteriorations/exacerbations | “Patients generally come back to consultations more often.”  “No reduced capacity, but much more severely ill patients.” |
| **Question (34)** | **What problems not mentioned here occurred during the pandemic, and what good practices not asked about here have worked well at your facility?** |
| **Problems** |  |
| Structural problems of the buildings | “Problematic structural conditions of the buildings with multi-bed rooms with shared wet rooms, rooms without wet rooms etc” |
| Shortage of hygiene and protection gear and equipment | “Availability of masks was difficult in the first wave”  “Bad: Supply of protective equipment until December 2020.” |
| Hygiene measures: time & effort | “Admission triage was problematic (staff-intensive, with no additional staff available).”  [Hygiene measures in mental health] “care are a considerable additional expense, especially in the inpatient sector.” |
| Implementation of hygiene measures in patients with mental disorders | „Difficult situation for staff in dealing with patients without insight into their illness (e.g., not wearing mask or not wearing mask correctly) with simultaneous increased risk of own SARS-CoV-2 infection.”  “Measures could be implemented well, but had very negative effects on atmosphere, less therapies offered."  "Hygiene plan was well adhered to, but had significant consequences on clinical care of pat. (esp. social psychiatric measures).” |
| Vaccination | “Problem: Vaccination prioritization too complicated” |
| Handling of exceptions | “Staff and patients brought many exception certificates (e.g. from mask requirement); difficult to deal with patients exceptions.” |
| Increasing burden on patients | "Problems: More frequent acute presentations due to psychosocial stress.”  “The reduction in the number of beds has significantly increased the pressure of admissions, which has had a particular impact on elective treatment options (e.g., specialized psychotherapy programs); in addition, there has been a shortening of stays as well as an increase in severity of illness of the patients” |
| Absenteeism of medical staff | “High workload of the nursing staff in the acute care unit and as a consequence absenteeism due to patients who are seriously ill for a long period of time”  “Increased absenteeism in the nursing sector, reasons: Fear of being deployed on infection units, domestic extra workload (family/alternating shifts), “substitution carousel”, poor communication/circulating misinformation.” |
| Demoralization | Very problematic for comittment of employees and patients in the psychosomatic and day clinic units: Self-image and esteem of employees severely affected by closing of wards.” |
| Political (mis)decisions: Changes of rules | “The frequent changes in rules regarding testing, etc. tended to push small facilities to the breaking point.” |
| Political (mis)decisions: Financial compensations | "[First pandemic wave:] Politically problematic decision [about compensation payments]: more money is earned via a vacant bed than via an occupied bed.”  “Inpatient Psychiatry in 2nd wave did not receive any compensation payments”  “Fear that at the end of the pandemic the administration/health insurance companies might say: "It was possible with much less capacity, we'll reduce your funding!”” |
| Political (mis)decisions: interinstitutional | "Information flow: Bottum-up – misinformation & scaremongering; Top-down –too late reaction with in the beginning intransparent information management. |
| Telemedicine | “Contact via. Telemedicine difficult for structurally disturbed patients and gerontopsychiatric patients.”  “In the case of severely ill patients, the technical prerequisites for telemedical services do not exist." |
| Reductions of services and offers | “The more chronically ill patients (disorder-specific therapy for borderline, PTSD, etc.) were probably severely unserved by closures of elective programs.” |
| Reductions of outpatient care structures | “Difficulties in placing patients in suitable forms of housing (assisted living, shared apartments, nursing homes)”  “Reduction of outpatient therapy services (day care centers, outpatient occupational therapy, etc.) leads to a noticeable increase in symptoms of patients in outpatient treatment.” |
| Lack of cooperation with outpatient sector | “No interaction/collaboration with outpatient system in region to ensure care despite reductions, no care coordination.”  “Patients reported even greater difficulties than usual in getting outpatient [mental health care] appointments, e.g. for psychotherapy.” |
| Impairment of development of services | “Constant crisis mode with regard to COVID-19 causes further development of care to stall.” |
| **Good-practice examples** |  |
| Leadership | “Permanent low-threshold accessibility of the head physician and head of the nursing service with regard to hygiene; thus high acceptance among employees; dare to ask questions” |
| Crisis management group | “Positive experiences with multi-professional weekly crisis-team-meeting”  “"A newly established "Corona Hospital Conference" that meets regularly has significantly improved interdisciplinary collaboration with somatic departments.” |
| Communication of hygiene measures | “Good communication with the physician in charge of hygiene (quick accessibility during office hours)”  “To improve communication, multipliers were used (also nursing) & number of meetings between different professional groups increased.”  “COVID-19 hotline of the central hygiene department for employees; comprehensive information material of the university hospital” |
| Support for employees | [More] “supervision/intervision opportunities for staff (were well used).” |
| Commitment | “Commitment of the employees was high.” |
| Cooperation within the institution | Improvement of the team spirit and cooperation with departments of other specialties |
| Cooperation within other institutions | [Improved] “Coordination with neighboring somatic hospitals (co-care of psychiatric patients by telephone)” |
| Care for patients with SMI and (potentially) SARS-CoV-2-infection | “Half of a ward converted to COVID area; patients allocated to this ward until negative PCR test was available, then distributed [to other wards]”  “On each ward one room [kept] free for isolation after admissions” |
| Care for patients with SMI and SARS-CoV-2-infection on internal medicine wards | “Transfer corridor to internal medicine (infectious diseases) created: PSY patients symptomatic of COVID-19 (fever, oxygen saturation <95%) transferred to Internal Medicine (COVID-19-ward); Internal Medicine patients with improved general condition and mental disorder transferred to PSY isolation ward in exchange.” |
| Contact reductions / Social distancing: medical staff | “Use of telemedicine for organizing exchanges (e.g., meetings; outpatient clinic meetings) -> travel time saved, calmer”; “Training via video conferencing”, “Shorter handovers, fewer meetings”, “no common meals of the staff” |
| Contact reductions / Social distancing: medical staff | “Group therapy: smaller groups, shorter times but more frequent” |
| COVID-19-Testing | “Acute presentations were swabbed in parallel both for rapid antigen testing and PCR testing, as long as their antigen test was negative, patient were allowed to move freely on ward”  “Two rapid inhouse PCR machines -> results available in approx. 30 minutes; if result positive, transfer to isolation ward; isolation in room until result available” |
| Vaccination | “Positive: mental health clinic was model project in which patients were vaccinated by the local staff" |
| Outpatient offers by the cinic | “Hometreatment/Inpatient-equivalent treatment is maintained in compliance with general hygiene rules and is a compensation option for lacking inpatient capacity” |
| Flexible introduction of new structures | “Technical enabling/creating options that were previously impossible for a long time, e.g. telemedicine, thus providing more flexible care.”  “WIFI for employees as well as for patients, to facilitate telemedical connection” |
| Technical Solutions: Telemedicine | “Telemedical offers are a good complement but less effective then in-person contacts.” |
| Teaching | “Online courses good complement to face-to-face teaching at the bedside." |
