# Supplementary material for: Changes and Challenges in Inpatient Mental Health Care During the First Two High Incidence Phases of the COVID-19 Pandemic in Germany – Results From the COVID Ψ Psychiatry Survey
Source: Front Psychiatry. 2022 Apr 27;13:855040. doi: 10.3389/fpsyt.2022.855040 (PMC9091906; doi:10.3389/fpsyt.2022.855040)
Supplement: Supplementary file 2 [file Table_2.DOCX]

**Guiding principles for the survey:**

1. Identification of the current state of mental health care in the pandemic.
2. Identification of good practice solutions in mental health care in the pandemic.

# General:

## How is the care profile of your clinic characterized (multiple answers possible):

□ Psychiatry and psychotherapy

□ Psychosomatic medicine and psychotherapy

□ specialised clinic

□ Department in a general hospital

□ Psychiatric care mandate

□ Clinic that offers emergency treatments

□ Clinic that offers elective treatments

□ Clinic that offers rehabilitation treatments

□ Clinic that offers inpatient treatments

□ Clinic that offers day-clinic treatments

□ Clinic that offers outpatient treatments (Institutsambulanz (PIA))

□ Other: _____________

## Please provide the first two digits of your hospital's zip code. (These data are used only for correlation with maximum regional COVID-19 incidence; no analyses are performed on individual hospitals).

# Inpatient/day-clinic capacities

## How did the total occupancy change during the pandemic for the individual diagnosis groups? This refers to the absolute numbers during the period of greatest pandemic burden across both pandemic waves (spring 2020 or turn of 2020/21) compared to the same period in 2019.

|  | Decrease > 20 % | Decrease > 10 % | Steady | Increase  > 10 % | Increase > 20 % | Generally not in the treatment spectrum |
| --- | --- | --- | --- | --- | --- | --- |
| F0: Dementias and delirium |  |  |  |  |  |  |
| F1:  Addictive disorders |  |  |  |  |  |  |
| F2:  Psychoses |  |  |  |  |  |  |
| F3: Affektive Disorders |  |  |  |  |  |  |
| F4:  Neurotic, Stress-related and somatoform disorders |  |  |  |  |  |  |
| F5:  Eating disorders |  |  |  |  |  |  |
| F6: personality disorders |  |  |  |  |  |  |

□ Question impossible to answer because figures not available.

## If you use the 2019 occupancy numbers as a comparison, what percentage of occupancy has been curtailed in the pandemic to date?

## The question again refers to the time when the pandemic burden was highest. Both waves (March/April 2020 and November/December 2020/January 2021) are to be recorded separately.

|  | 0% | 10% | 20% | 30% | 40% | 50% | 60% | 70% | 80% | 90% | 100% |
| --- | --- | --- | --- | --- | --- | --- | --- | --- | --- | --- | --- |
| Minimum of the first wave (spring 2020) Inpatient |  |  |  |  |  |  |  |  |  |  |  |
| Minimum of the first wave (spring 2020) Day-clinic |  |  |  |  |  |  |  |  |  |  |  |
| Minimum of the first wave (spring 2020) Outpatient |  |  |  |  |  |  |  |  |  |  |  |
| Minimum of the second wave (turn of the year 2020/2021) Inpatient |  |  |  |  |  |  |  |  |  |  |  |
| Minimum of the second wave (turn of the year 2020/2021) Day-clinic |  |  |  |  |  |  |  |  |  |  |  |
| Minimum of the second wave (turn of the year 2020/2021) Outpatient |  |  |  |  |  |  |  |  |  |  |  |

□ Question impossible to answer because figures not available.

## Why was the occupancy restricted as indicated in question #4 - if this was the case *(Multiple selection possible)*

|  | The offer was scaled back as a general protective measure on the part of the clinic | The offer was reduced in order to be able to maintain social distancing (e.g., four-bed rooms dissolved, isolation rooms created) | The offer was cut back due to staff shortages | Services were scaled back because staff had to be assigned to care for COVID-19 patients | Demand on the part of the patients decreased | Other reasons |
| --- | --- | --- | --- | --- | --- | --- |
| First wave (spring 2020) Inpatient |  |  |  |  |  |  |
| First wave (spring 2020) Day-clinic |  |  |  |  |  |  |
| First wave (spring 2020) Outpatient |  |  |  |  |  |  |
| Second wave (winter 2020/2021)  Inpatient |  |  |  |  |  |  |
| Second wave (winter 2020/2021)  Day-clinic |  |  |  |  |  |  |
| Second wave (winter 2020/2021)  Outpatient |  |  |  |  |  |  |

**Question #5b: What other reasons were there for limiting occupancy?**

_________________________________________________

Free text (question to be answered only if "other reasons" indicated in previous question)

## How did the types of admission have changed during the pandemic?

|  | Increase | Decrease | Steady | Not part of the treatment spectrum |
| --- | --- | --- | --- | --- |
| Elektive admissions (nach Warte-liste) |  |  |  |  |
| Emergency admissions without acute risk |  |  |  |  |
| Emergency admissions with acute risk |  |  |  |  |

## What difficulties were encountered due to reduced inpatient and day-clinic care in the two waves of the pandemic? *(Multiple selection possible)*

□ No difficulties

□ Increase of admissions after the first wave (spring 2020)

□ Lack of integration into the living environment at the end of treatment

□ Deteriorations, exacerbations or relapses. In addictive disorders

□ Suicide attempts/suicides

□ Contact breakdowns

□ Other: _________________________

## What difficulties were encountered due to reduced inpatient and day-clinic care in the two waves of the pandemic? *(Multiple selection possible)*

□ No difficulties

□ Increase of admissions

□ Lack of integration into the living environment at the end of treatment

□ Deteriorations, exacerbations or relapses. In addictive disorders

□ Suicide attempts/suicides

□ Contact breakdowns

□ Other: _________________________

## During the two waves of the pandemic, how were patients cared for who were discharged early or who were not admitted because of the protective measures but who needed treatment ?

□ By outpatient psychotherapists

□ By outpatient psychiatrists/neurologists

□ By the clinics/departemnts outpatient services (psychiatrische Institutsambulanz)

□ By telemedicine

□ Not at all

□ I do not know

□ Other: ____________

## If your facility provides inpatient-equivalent home treatment, has this been continued?

□ Yes, it has been continued

□ No, it was paused as a protective measure for patients and employees

□ No, it was paused at the request of the patients

□ Not on offer in our facility

## Do you have concerns about potential financial losses due to reduced occupancy in the pandemic? *(Multiple selection possible)*

□ Yes

□ No, no concerns because of compensation payments or other offsets in the first wave (spring 2020).

□ No, no concerns because of compensation payments or other offsets in the second wave (winter 2020/2021).

□ No, overall no problem in the first wave (spring 2020).

□ No, no problem overall in the second wave (winter 2020/2021)

**11a. If so, what are your concerns about potential financial losses due to reduced occupancy in the pandemic?**

□ Concerns about lack of compensation or other offsets in the first wave (spring 2020).

□ Concerns about lack of compensation or other offsets in the second wave (winter 2020/2021).

□ Other concerns: _________________________________

# Inpatient/Day-clinic hygiene

## Was a pandemic plan in place at the beginning of the pandemic? If not, when was it introduced?

□ It was in place before the pandemic

□ No, it was not introduced either.

□ No, but it was introduced on ... (please enter date in the comment field!) __________________

## How could the following measures be implemented at your facility during the pandemic?

|  | Very problematic | Problematic | Neutral | Good | Very good | Not applicable |
| --- | --- | --- | --- | --- | --- | --- |
| Mssk mandate |  |  |  |  |  |  |
| Social distancing |  |  |  |  |  |  |
| Periodic hand sanitizing |  |  |  |  |  |  |
| Reduced room occupancy |  |  |  |  |  |  |
| Periodic testing of staff and patients |  |  |  |  |  |  |
| Spatial separation during meals |  |  |  |  |  |  |
| Pausing group therapies |  |  |  |  |  |  |
| Reduced occupancy of therapy groups |  |  |  |  |  |  |
| Visiting restrictions |  |  |  |  |  |  |
| Restrictions of overnight stay-at-homes |  |  |  |  |  |  |
| Restrictions of visits at home |  |  |  |  |  |  |
| Restriction on leaving the hospital |  |  |  |  |  |  |

## What kind of COVID-19 screening or testing do you currently use for new admissions?

□ Screening by questionnaire and temperature measurement

□ Rapid antigen test

□ PCR test

□ None of the above methods

# Management of SARS-CoV-2 positive patients with leading mental illness.

## What is the total number of SARS-CoV-2 positive patients you have had at your facility?

□ 0

□ 1-10

□ 11-20

□ >20

If you had SARS-CoV-2 positive patients* in your facility, how many at a time? And what percentage of total occupancy did this represent? Please enter in the comments field: ____________________________

## Have there been SARS-CoV-2 outbreaks in wards at your facility.?

□ Yes

□ No

## Wehat measures are available to treat patients* with COVID-19 at your facility? ? *(Multiple selection possible)*

□ O2-Supply

□ Monitor

□ rooms usable as "airlock"

□ None of the above

□ No indication

## Is there a manual (standard operating procedures) for the diagnosis and treatment of patients with a leading mental illness and concurrent SARS-CoV-2 infection in your institution??

□ Yes

□ No

□ I do not know

## Have patients been accommodated and treated at your facility during the pandemic under the Infection Protection Act (IfSG)??

□ No

□ Yes, SARS-CoV-2 positive patients* with leading mental illness.

If yes: Number:

□ Yes, SARS-CoV-2 positive patients* without leading mental illness.

If yes: Number:

## Were SARS-CoV-2-positive patients coercively accommodated and treated in your facility during the pandemic under the usual mental health acts for endangerment of self or others (“Landesgesetze über Hilfen bei psychisch Erkrankungen PsychKHG/PsychKG” or “Bundesgesetzbuch BGB”)

□ No

□ Yes

If yes: Number:

## Have any patients (with or without SARS-CoV-2 infection) been presented to your facility for noncompliance with hygiene or quarantine rules who did not otherwise have an acute mental illness requiring treatment??

□ No

□ Yes, patients with such case constellations were presented by the authorities but not admitted

□ Yes, patients with such case constellations were presented by the authorities and admitted. Please specify quantity: _____________

## How were patients treated in your catchment area with a SARS-CoV-2 infection who had a leading mental illness resulting in the need for inpatient treatment?

|  | Applied and positive experiences | Applied and negative experiences | Did not apply |
| --- | --- | --- | --- |
| Care in isolation rooms/areas on regular wards in psychiatric/psychosomatic clinic/department |  |  |  |
| Care in own psychiatric COVID-19 ward in psychiatric/psychosomatic clinic/department without consultative internal medicine services |  |  |  |
| Care in own psychiatric COVID-19 ward in psychiatric/psychosomatic clinic/department with consultative internal medicine services |  |  |  |
| Care on general internal medicine infection ward without consultative psychiatric co-treatment |  |  |  |
| Care on overlapping internal infection ward with consultative psychiatric co-treatment |  |  |  |

## Is there a dedicated COVID 19 station at your facility?

□ Yes

□ No

## What was your experience with internal medicine co-treatment of SARS-CoV-2 positive psychiatric patients?

|  | Positive | Negative | Did not apply |
| --- | --- | --- | --- |
| Consultative internal medicine co-treatment on site |  |  |  |
| Consultative internal medicine co-treatment by telephone/video |  |  |  |

## Have there been any problems with transfer to COVID-19 internal medicine wards?

□ No, there were no problems.

□ Yes, there were problems: The internal medicine colleagues questioned our assessment of the patients as internally critical.

□ Yes, there were problems: The internal medicine colleagues overestimated the possibilities of psychiatry/psychosomatics to treat COVID-19 patients.

□ Yes, there were problems: The internal medicine facilities/wards had no capacities.

□ Yes, there were problems: there were reservations and fears about COVID-19 patients with additional mental illness

□ No such transfer has taken place to date.

# Personell

## Have there been SARS-CoV-2 infections among (medical and non-medical) staff at your facility?

□ Yes

□ No

**26a. What was the maximum level of staff absences??**

| 0% | 10% | 20% | 30% | 40% | 50% | 60% | 70% | 80% | 90% | 100% |
| --- | --- | --- | --- | --- | --- | --- | --- | --- | --- | --- |
|  |  |  |  |  |  |  |  |  |  |  |

## Did SARS-CoV-2 infected employees cause further infections among employees??

□ Yes

□ No

□ Not assessable

## Did you have difficulty recruiting staff to care for SARS-CoV-2 positive patients during the first pandemic wave (spring 2020)?

|  | Yes | No | Not applicable |
| --- | --- | --- | --- |
| It was difficult to recruit ***medical*** staff for this. |  |  |  |
| It was difficult to recruit ***nursing*** staff for this. |  |  |  |
| It was difficult to recruit ***support staff*** *(room maintenance, student assitants etc.)* for this. |  |  |  |
| There was increased absenteeism compared to the average among ***medical staff*** assigned to it |  |  |  |
| There was increased absenteeism compared to the average among ***nursing staff*** assigned to it |  |  |  |
| There was increased absenteeism compared to the average among ***support staff*** *(room maintenance, student assitants etc.)* assigned to it |  |  |  |

## Did you have difficulty recruiting staff to care for SARS-CoV-2 positive patients during the second pandemic wave (winter 2020/2021)?

|  | Yes | No | Not applicable |
| --- | --- | --- | --- |
| It was difficult to recruit ***medical*** staff for this. |  |  |  |
| It was difficult to recruit ***nursing*** staff for this. |  |  |  |
| It was difficult to recruit ***support staff*** *(room maintenance, student assitants etc.)* for this. |  |  |  |
| There was increased absenteeism compared to the average among ***medical staff*** assigned to it |  |  |  |
| There was increased absenteeism compared to the average among ***nursing staff*** assigned to it |  |  |  |
| There was increased absenteeism compared to the average among ***support staff*** *(room maintenance, student assitants etc.)* assigned to it |  |  |  |

## What additional ways of communicating with employees* were added during the pandemic? *(Multiple selection possible)*

□ Telephone conferences

□ Video-Meetings

□ E-mail newsletters

□ Information events with multipliers (analog or digital)

□ No changes

□ Other ways of communication: _____________________________

## In which prioritization group for COVID-19 vaccination is the staff at your clinic?

|  | Highest priority (Gruppe 1) | High priority (Gruppe 2) | Neither group 1 nor 2 or unclear |
| --- | --- | --- | --- |
| Medical staff (including physicians, nurses, psychotherapists) with contact to SARS-CoV-2 positive patients |  |  |  |
| Medical staff (including doctors, nurses, psychotherapists) with contact to patients who cannot comply with the protective measures. |  |  |  |
| Medical staff (including doctors, nurses, psychotherapists) with contact to patients who largely comply with the protective measures. |  |  |  |
| Non-medical staff with patient contact (e.g., reception, transport service, room care). |  |  |  |
| Non-medical staff without patient contact (e.g. scientists, administration, technical assitants) |  |  |  |

# Telemedicine:

## Which telemedicine offerings were used at your facility before the pandemic, which were newly introduced during the pandemic, and for which are you planning to continue using them? (*Multiple selection possible* *but please select only meaningful answer combinations)*

|  | not in use | already in use before the pandemic | newly introduced during pandemic | continued use after pandemic planned |
| --- | --- | --- | --- | --- |
| Telephone consultation |  |  |  |  |
| Video consultation |  |  |  |  |
| Selfhelp-Apps |  |  |  |  |

## For which groups of patients were telemedicine services used during the pandemic and what was the experience?

|  | F0: Dementias and Delirium | F1: Addiction disorders | F2:  Psychoses | F3: Affective disorders | F4: Neurotic, stress-associated, somatoform | F5:  Eating disorders | F6: Personality disorders |
| --- | --- | --- | --- | --- | --- | --- | --- |
| Not used |  |  |  |  |  |  |  |
| Good experiences |  |  |  |  |  |  |  |
| Problematic experiences |  |  |  |  |  |  |  |
| No answer |  |  |  |  |  |  |  |

# Final questions

## What problems not mentioned here occurred during the pandemic, and what good practices not asked about here have worked well at your facility?

_________________________________________________

(Free text)

## Was this survey conducted in an interview or without an interview?

□ With interview

□ Without interview
